# Improved Margins Detection of Regions Enriched with Gold Nanoparticles inside Biological Phantom

**DOI:** 10.3390/ma10020203

**Published:** 2017-02-20

**Authors:** Yossef Danan, Inbar Yariv, Zeev Zalevsky, Moshe Sinvani

**Affiliations:** 1 Faculty of Engineering, Bar-Ilan University, Ramat-Gan 5290002, Israel; inbarbossi@gmail.com (I.Y.); zeev.zalevsky@biu.ac.il (Z.Z.); sinvanm@gmail.com (M.S.); 2 The Bar-Ilan Institute of Nanotechnology & Advanced Materials, Bar-Ilan University, Ramat-Gan 5290002, Israel

**Keywords:** surface plasmon resonance, gold nanorods, photothermal imaging, laser beam modulation

## Abstract

Utilizing the surface plasmon resonance (SPR) effect of gold nanoparticles (GNPs) enables their use as contrast agents in a variety of biomedical applications for diagnostics and treatment. These applications use both the very strong scattering and absorption properties of the GNPs due to their SPR effects. Most imaging methods use the light-scattering properties of the GNPs. However, the illumination source is in the same wavelength of the GNPs’ scattering wavelength, leading to background noise caused by light scattering from the tissue. In this paper we present a method to improve border detection of regions enriched with GNPs aiming for the real-time application of complete tumor resection by utilizing the absorption of specially targeted GNPs using photothermal imaging. Phantoms containing different concentrations of GNPs were irradiated with a continuous-wave laser and measured with a thermal imaging camera which detected the temperature field of the irradiated phantoms. By modulating the laser illumination, and use of a simple post processing, the border location was identified at an accuracy of better than 0.5 mm even when the surrounding area got heated. This work is a continuation of our previous research.

## 1. Introduction

The ability to resect a tumor completely is a key merit in preventing the recurrence of the disease. In order to achieve more complete tumor resection, the surgeon must clearly identify the tumor margins. This identification is even more crucial when the tumor growth is adjacent to or in neurological structures, and therefore it is dangerous to remove extra tissue [1,2]. Various methods have been proposed to better visualize tumor margins. Among these methods are magnetic resonance imaging (MRI) [2], CT [3] and targeted fluorescence imaging [4]. However, MRI and CT, because of their prolonged process, suffer from limited spatial resolution due to tissue shift during surgery [5], and targeted fluorescence imaging depends on the photophysics or photochemistry of the fluorophore, the autofluorescence of live cells, photo toxicity and photo bleaching [6,7,8,9,10].

Gold nanoparticles (GNPs) have been used for the last two decades by numerous research groups as biomarkers in multiple biological applications such as drug delivery [11], imaging contrast agents [12,13] and therapeutics [14,15]. Important attributes of GNP are their photostability and biocompatibility [16]. The main interest in GNPs stems from the surface plasmon resonance (SPR) that results in high scattering and absorption cross-sections [17]. When the GNPs are illuminated by a wavelength that matches the SPR, a strong oscillating motion of the electrons in the GNPs will occur, resulting in amplification of their optical absorption and scattering [18,19]. The resonance wavelength depends on the GNP’s dimensions and the refractive index of the nanoparticle and its environment [17]. The light scattering is essential for imaging applications based on light-scattering modalities, including dark field microscopy [20,21] or coherence tomography [22,23]. The approach based on light absorption is used, for instance, for bright field microscopy and photothermal therapy [24,25,26]. In photothermal therapy, the strong absorption of the GNPs is utilized to elevate the temperature to at least 50 °C in order to achieve effective denaturation of proteins and cell death [27].

The method proposed in this paper is a photothermal imaging using modulated laser beam radiation on targeted gold nanorods (GNRs) and a thermal camera. By illuminating the GNRs with a wavelength corresponding to their SPR, the GNRs absorb optical energy which turns in to heating of the GNRs, which spread to their environment. These particles can be specifically targeted to decorate the surface of cancer cells [28,29,30]. Thus, the temperature elevation occurs inside the cancerous tissue, enabling us to distinguish between cancerous and noncancerous areas by using a thermal camera. In our previous study we showed the basics of detecting tumor borders using the absorptions of GNPs and a photothermal camera [31]. However, because a continuous-wave (CW) laser was used, the temperature was elevated continually and dissipated out of the GNPs’ area, and thus the border detection was less accurate. In this paper, to maximize the distinction of the tumor margins, a modulation of the laser was applied to minimize heat dissipation to noncancerous surrounding tissue. Moreover, by subtraction between the thermal images of the maximum and minimum temperature in each thermal cycle, improvement of the border detection spatial resolution and the signal-to-noise ratio (SNR) was achieved, even when the overall temperature was elevated. Using photothermal imaging instead of the widely-used imaging applications based on light-scattering modalities allowed us to prevent the background noise caused by light scattering from the tissue, thus improving the SNR and achieving higher contrast between the target cancerous cells and the healthy tissue.

## 2. Experimental Setup

**Figure 1:**
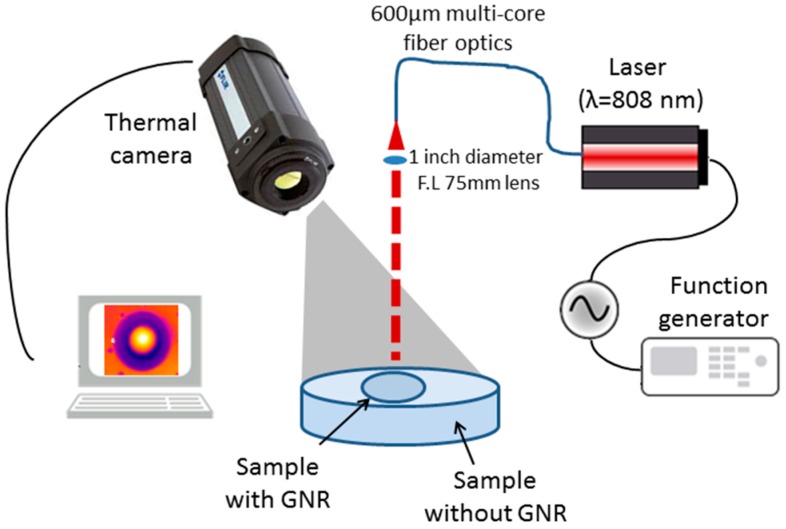
The experimental setup. GNR: gold nanorod.

The experimental setup was design to image the temperature distribution over the sample under laser irradiation by a radiometric thermal imaging camera (FLIR Systems Inc., model A325, Wilsonville, OR, USA). As shown in Figure 1, the laser illuminates the sample from above, in perpendicular to the sample surface. A near infra-red (NIR) laser at wavelength of 808 nm and power density of 1.6 W/cm^2^ and less was used. This NIR laser is in wavelength near the longitudinal SPR of the GNRs we used. The NIR region of the spectrum provides the maximal penetration depth of light into the biological tissue. The penetration depth of red and NIR light, in this region between 650 and 900 nm, is up to 100 mm, depending on the exact type of tissue [32]. In comparison, the penetration depth of green Nd:YAG laser (532 nm wavelength) is less than 0.5 mm [32]. The laser illumination was modulated using a function generator (AFG3022B by Tektronix, Beaverton, OR, USA) to create a square wave, where the rise time and the fall time are short compared to the on time of a period of the laser pulses, with different frequencies and duty-cycles.

The temperature change over the sample was imaged using the thermal camera which provides the temperature field of the image. The camera has a temperature sensitivity of 0.07 °C and 320 × 240 pixels. The spatial resolution of the camera is 0.5 mm for one pixel. This camera sensitive to thermal radiation at a wavelength region of 8–14 μm, and thus is completely blind to other light sources including the laser illumination.

## 3. Results and Discussion

### 3.1. Solid Phantom Preparation

Solid tissue–like phantoms were prepared for simulating the optical properties of tissues [33]. The solid phantoms were prepared in a few phases in order to create phantoms enriched by GNRs surrounded by a reference phantom without GNRs. First, the inside phantoms (30 mm diameter) were prepared by adding different concentrations of GNRs (0.025, 0.05, 0.07, 0.1 mg/mL). Following solidification, the inside phantoms were transferred into 90 mm cell culture plates, where the background reference phantom solution was poured carefully around them to create a uniform upper surface with a direct interface between the phantoms with and without GNRs. Finally, the combined phantoms were cooled under vacuum conditions to avoid bubbles.

The phantoms were prepared using 2% IntraLipid (IL) (IntraLipid 20% Emulsion, Sigma-Aldrich, Rehovot, Israel) as a scattering component, 1 × 10^−3^% India Ink as an absorption component [34], double distilled water (DDW) and 1% agarose powder (SeaKem LE Agarose, Ornat, Rehovot, Israel) in order to convert the solution into a gel. The solutions were heated and mixed (at a mixing temperature of 90 °C) while the agarose powder was slowly added. The phantoms were prepared in cell culture plates (30 and 90 mm) and were cooled under vacuum conditions to avoid bubble formation.

### 3.2. Gold Nanorod (GNR) Characterization

**Figure 2:**
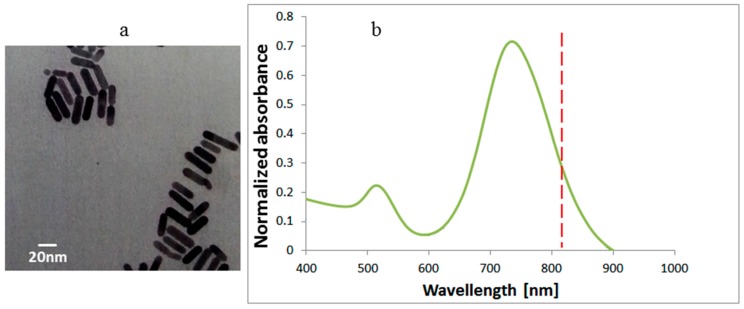
(**a**) Transmission Electron Microscope (TEM) image of the GNRs with 37 nm length and 10 nm diameter and (**b**) normalized the absorbance spectra of the GNRs. The laser wavelength is shown by a red dashed line.

The gold nanorods used had a 37 nm length and a 10 nm diameter, purchased from Nanopartz Inc. (Loveland, CO, USA). In Figure 2a, one can see the TEM (Transmission Electron Microscope) (CM 100, Philips, Eindhoven, The Netherlands) image of the GNRs and the absorbance spectrum (ultraviolet-visible spectrometer, Shimadzu, UV1650 PC, Tokyo, Japan). The peak at 530 nm arises from the electron’s resonance at the small size (10 nm) of the GNRs and the peak at 770 nm arises from the long size (37 nm) resonance.

### 3.3. Experimental Results

**Figure 3:**
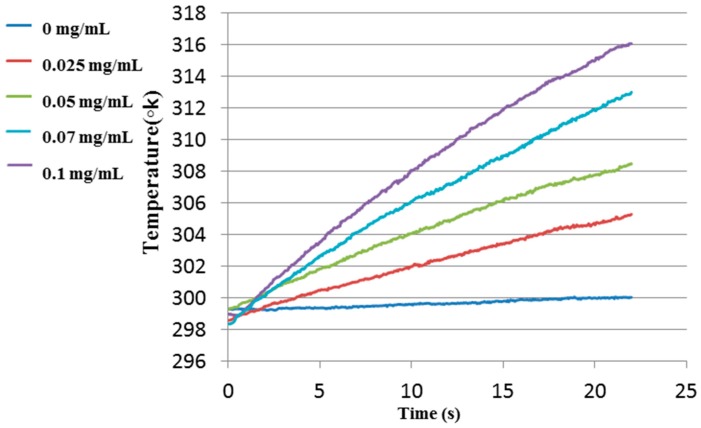
The temperature elevation as a function of illumination time using 808 nm laser for phantom with different concentrations of GNRs.

In order to examine the heating profile of the phantoms, they were irradiated with a CW laser at a 808 nm wavelength and the temperature change at the beam center was recorded until reaching about 43 °C for the highest concentration of 0.1 mg/mL. The illumination was stopped at this temperature in order to prevent damage to the phantoms. Figure 3 shows the temperature elevation profiles for the irradiated phantoms enriched with GNRs as a function of time for different GNR concentrations.

**Figure 4:**
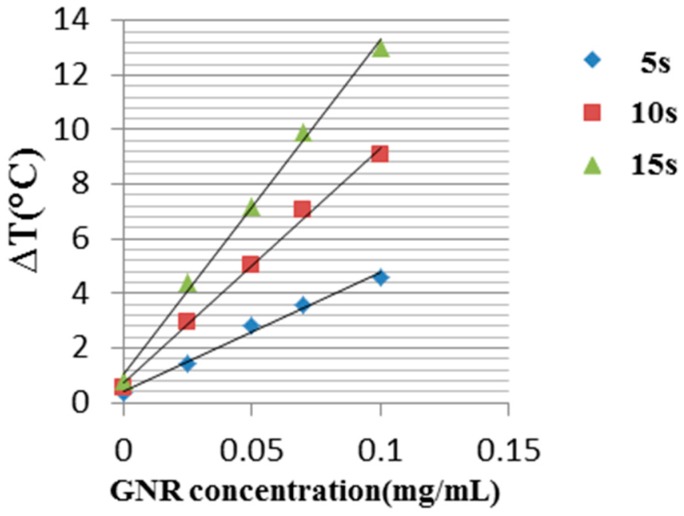
Temperature change as a function of GNRs concentration for three different illuminations times: 5, 10 and 15 s.

As shown in Figure 3, the temperature of the sample without GNRs remained almost unchanged, while the temperatures of the samples with GNRs were elevated with the heating time. The relatively small temperature change of the reference phantom was due to the India Ink that was added to the phantoms as an absorption component to mimic the natural absorption of healthy tissue. Furthermore, it demonstrated that there is a correlation between the GNR concentration and the temperature elevation. For the highest GNR concentration (0.1 mg/mL), after 20 s, a temperature elevation of about 16 °C was observed, while for the lowest concentration (0.025 mg/mL), the temperature change after 20 s was about 6 °C. In Figure 4 the linear temperature changes as a function of the GNR concentration are shown. One can see a linear relation between the GNR concentration and the temperature elevation.

**Figure 5:**
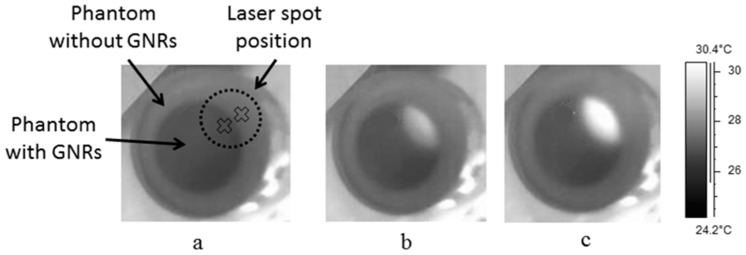
Thermal images of the sample: (**a**) without irradiation; (**b**,**c**): after 5 and 10 s of continuous-wave (CW) illumination, respectively. It can be seen that the heated zone increased during the illumination time.

In order to improve the exact border detection, the laser illumination was modulated using a function generator (AFG3022B by Tektronix). The modulated signal was a square wave with a frequency of 0.1 Hz and different duty-cycles. Figure 5 shows the temperature change as a function of time when the illumination was on the border between the areas with and without GNRs in the phantom. The temperature was measured at equal distances from the beam center, which was located on the border, towards the two sides of the phantom, to simultaneously compare the temperature rise on both sides of the border. The exact location of the points on the sample, where the temperature was measured, is illustrated in Figure 5a by a cross on each side of the border.

In Figure 5b,c we can see the thermal images after 5 and 10 s of irradiation, where the GNRs’ phantom region and the surroundings without the GNRs are easily distinguishable. We also can see that the heated zone was increased during the illumination time and caused the movement of the border seen in the photothermal images, taken in situ, due to continuous heat generation by the laser irradiation absorbed by the GNRs and dissipated to the surrounding phantom with no GNRs. To overcome this unwanted effect, we modulated the laser beam at a frequency of 0.1 Hz in a duty-cycle of 5% in one case. The importance of the pulsed-like laser irradiation is that even if the heat from the GNR area diffused to the surroundings and smeared or moved the border line, on the thermal image, each laser pulse shows again the exact location of the border on top of the global rise of the temperature. By the use of a simple image processing algorithm, the effect of the global heating is removed as will be shown later on. Also, it is obvious that the pulsed laser radiation decreased the total power absorbed by the factor of the duty cycle.

**Figure 6:**
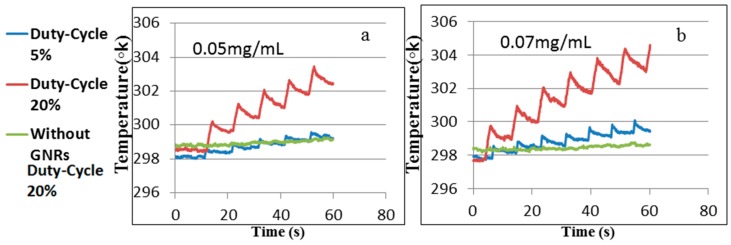
Temperature change as a function of time modulated by laser illumination for two different GNR concentrations: (**a**) 0.05 mg/mL; and (**b**) 0.07 mg/mL.

In the experiment, the temperature change was measured on the two sides of the border, as shown on Figure 5a for two different GNR concentrations of 0.05 and 0.07 mg/mL. Two different duty-cycles were used, 5% and 20%, in order to demonstrate the differences in the heating and cooling processes, as shown in Figure 6.

In Figure 6, one can see that when the duty-cycle is longer, the temperature elevation is higher, where the temperature change due to the illumination absorption in the phantom without GNRs is negligible. Therefore, the major factor in the experiment is the very high absorption of the areas enriched with GNRs compared to the absorption of areas without GNRs. 

**Figure 7:**
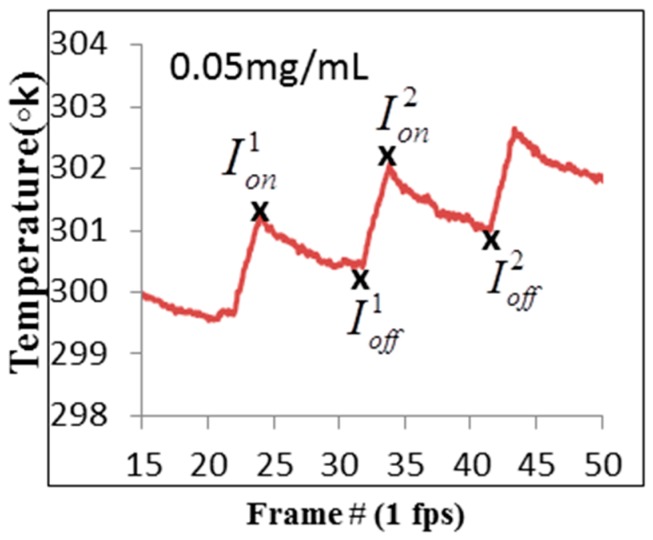
Temperature change as a function of frame number for GNR concentrations of 0.05 mg/mL and 20% duty-cycle laser illumination. The specific frames that were used in the proposed method are marked with a black **x**.

In order to improve the border detection spatial accuracy, a record of thermal images was taken during the modulated laser irradiation cycles at the frequency of 1 Hz. In each cycle we took 10 images simultaneously with the temperature records shown in Figure 6. The subtraction between the thermal images for the maximum and minimum temperatures in two successive thermal cycles was done as follows:

(1) $$I_{difference}(x,y)=I_{on}(x,y)-I_{off}(x,y)$$

where $I_{difference}$ is the difference in the image intensity and $I_{on}$ and $I_{off}$ are the image intensities for the maximum and minimum temperatures, respectively. The exact frames that were taken are shown in Figure 7.

**Figure 8:**
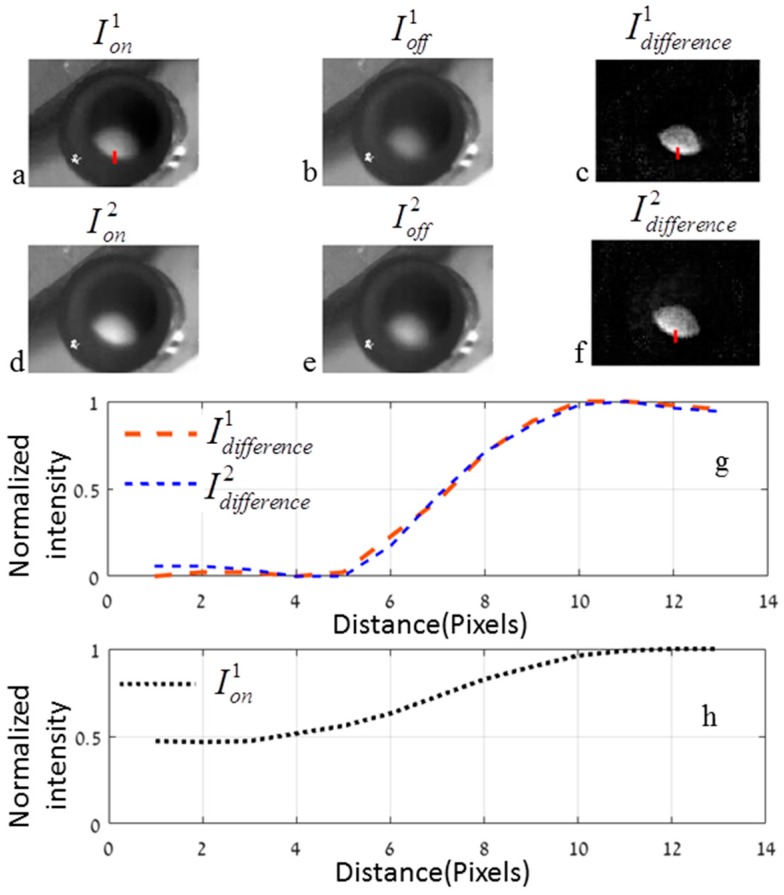
(**a**,**b**) and (**d**,**e**) Thermal images of the maximum and minimum temperature for two successive cycles; (**c**,**f**) Subtractions between the maximum and minimum in each cycle; (**g**,**h**) Intensity profile of the cross-sections marked with red lines in (**c**,**f**) and (**a**), respectively.

Figure 8a,b and Figure 8d,e show the thermal images’ maximum and minimum temperatures for two successive cycles, and the subtractions are shown in Figure 8c,f. One can see that after the subtractions, only the regions where the temperature was changed were left. These regions are exactly where the irradiation meets the GNRs, leading to better visualization of the phantom with the GNR border.

Shown in Figure 8g,h is the intensity profile of the cross-sections marked with red lines in Figure 8c,f and Figure 8a, respectively. One can see that the slopes of the two cross-sections’ intensities after subtraction are at the same pixels, and thus the overall heating of the phantoms due to the continual irradiation do not influence the accuracy of the exact border detection by using the suggested method. Moreover, by using the suggested method we canceled the heat dissipation effect on the border detection, and thus the heat diffusion coefficient does not play significant role. The same slope before the subtraction (shown in Figure 8h) extends on a larger number of pixels than after the subtraction, which means the resolution of the exact border was improved by using the proposed method. Moreover, the constant values were removed, leading to better visualization after the subtraction. The spatial resolution of the thermal camera was 0.5 mm, and therefore the proposed method can differentiate between areas with and without GNRs better than 0.5 mm even when the surrounding area is heated. By adding an extra lens, the thermal camera can achieve, at a working distance of 80 mm, a spatial resolution of 0.1 mm. The major limiting factors in the suggested method are the camera resolution and the temperature gradient in the boundary due to the GNR concentration gradient in the boundary. Because of the two-phase preparation process of the samples (Section 3.1), there is a diffusion of GNRs to the surroundings. As shown in Figure 8g,h, there is a temperature gradient in the boundary, which is in correspondence to the GNR gradient. Moreover, this gradient could mimic the state of a real tumor, where the concentration of the cancerous cells is less dense in the boundaries. However, the overall temperature elevation is canceled and the temperature gradients for different illumination cycles are identical. The doctor's need for tumor boundary detection accuracy varies for different types of tumors and their locations. In general, removing extra tissue reduces the reoccurrence of the disease [2,3]. However, exact tumor border identification is most crucial when the tumor growth is adjacent to or in neurological structures, and therefore it is dangerous to remove extra tissue. The exact resolution that the doctors can achieve nowadays during operations is in the range of a few millimeters [35]. However, special techniques have been developed in order to achieve sub-millimeter resolution [36,37]. Our method is intended to be better than these techniques. However, for post-operation methods, a resolution of a few microns and even smaller can be achieved by nano-microscopy using targeted GNPs in cancerous cells [13,38].

In real tumors the spatial distribution and the position of the exact border could be varied for different types of cancer, in contrast to the usually static GNR concentration in phantoms. Moreover, in real tissues some GNRs may be in non-cancerous tissues. Thus, the spatial transition area in real cases is less sharp in comparison to the studied phantom case. However, we can utilize our spatial separation ability to calibrate the GNR concentration threshold for different tumor types and then a thermal threshold can be set to obtain the maximal spatial diagnosis ability for the tumor’s border.

## 4. Conclusions

In this research we demonstrate the feasibility to identify the border of phantoms enriched with GNRs from their surroundings with an accuracy of better than 0.5 mm using modulated laser radiation and a thermal imaging camera. We demonstrated an image processing algorithm developed to isolate the GNR area from its surroundings accurately even under elevated temperatures. The lateral accuracy of the border detection can be achieved in laser power density lowered by a factor of ~5 compared to the 1.6 W/cm^2^ we used in this research, if the laser wavelength will be adapted to the GNRs’ resonance or alternatively to reduce the GNR concentration. Our laser wavelength is not exactly at the spectral location of the GNRs’ absorption peak, as shown in Figure 2b. These particles can be specifically targeted to decorate the surface of cancer cells. Thus, the temperature elevation occurs in the cancerous tissue, enabling us to distinguish between cancerous and non-cancerous tissues. An essential advantage of the proposed technique is the use of the absorption properties of GNPs rather than their scattering properties, leading to high contrast between targeted cancer cells and normal background tissue. We expect this in vitro technique will eventually lead to intraoperative photothermal imaging which will assist surgeons in determining tumor margins accurately during surgery, leading to complete tumor resection and improved patient outcome with a small concentration of GNPs and lower laser flounce.
